# Whole-Plant Rape Silage-Based Diets for Chongming White Goats: An Integrated Assessment of Growth Performance, Meat Quality and Gut Microbiota

**DOI:** 10.3390/foods14203512

**Published:** 2025-10-15

**Authors:** Rongrong Liao, Changfeng Xiao, Yuhua Lv, Yue Liu, Yuexia Lin, Lihui Zhu

**Affiliations:** Institute of Animal Husbandry and Veterinary Science, Shanghai Academy of Agricultural Sciences, Shanghai 201106, China; liaorongrong@saas.sh.cn (R.L.); xiaochangfeng@saas.sh.cn (C.X.); lvyuhua@saas.sh.cn (Y.L.); liuyue@saas.sh.cn (Y.L.)

**Keywords:** whole-plant rape silage, goat, meat quality, amino acids, intestinal health

## Abstract

Forage rape (*Brassica napus* L.) is increasingly becoming a valued forage choice in livestock production. However, research on the application of whole-plant rape silage (including pod shells) in goats remains limited. To evaluate the effects of whole-plant rape silage on goat growth performance, meat quality, antioxidant capacity, and intestinal health, a 90-day feeding trial was performed using 36 healthy 6-month-old Chongming white goats. The goats were fed ad libitum and divided into two groups: a control group (CON, *n* = 18) fed corn straw silage and a treatment group (TRT, *n* = 18) fed a diet containing a 1:1 mixture of whole-plant rape silage and corn straw silage. Results showed that a 50% substitution with whole-plant rape silage increased carcass weight (*p* = 0.005), enhanced total antioxidant capacity (*p* < 0.01) in plasma, reduced bitter amino acids (such as leucine, isoleucine and phenylalanine; *p* < 0.05) in muscle, promoted intestinal villi proliferation (*p* < 0.05), and increased the abundance of beneficial bacteria involved in carbohydrate metabolism (e.g., *Family*_*XIII*_*AD3011*_group; *p* = 0.028) and propionic acid metabolism (e.g., *Phascolarctobacterium*; *p* = 0.026). In conclusion, our findings demonstrated that whole-plant rape silage can serve as a viable alternative to corn straw silage for Chongming white goats.

## 1. Introduction

As China’s economy continues to grow rapidly, the demand for “low cholesterol, high protein” mutton is increasing year by year [1,2]. China has surpassed the United States to become the world’s largest meat consumer market and has ranked first in the world for mutton production and consumption for many years [3]. However, the shortage of high-quality forage grass in China, especially green feed in winter and spring, has become a key bottleneck restricting the quality and efficiency of the mutton industry and food safety. Roughage is the main component of goat diets, which has multiple functions of stimulating regurgitation, promoting saliva secretion and providing energy [4]. As the primary silage crop in China, maize is widely cultivated. However, its long-term monocropping leads to soil nutrient depletion and increases the risk of soil erosion [5]. To address this deficit, winter crop forage rape (*Brassica napus* L.) serves as a high-quality straw silage, rich in crude protein (>5.24%), vitamins, unsaturated fatty acids, and minerals, making it a potential feed resource for livestock [6,7,8,9]. Specifically, elevated inclusion rates of rape silage linearly increased muscle α-linolenic acid and essential amino acid concentrations in Hu lambs [9]. In contrast to corn straw silage, silage made from whole-plant rape considerably improved the apparent digestibility of dry matter, organic matter, crude fiber, and nitrogen in buffalo models. Additionally, it increased the levels of milk protein, milk fat, and total milk solids at the same time [8]. Furthermore, prolonged feeding of forage rape significantly reduced methane emissions from sheep while increasing ruminal propionate, succinate, and primary alcohols, thereby demonstrating dual benefits of emission mitigation and production efficiency [10,11,12]. A recent study on dairy buffaloes indicated that feeding whole-plant rape silage increased milk fat and protein content but did not affect milk yield, lactose content, or urea nitrogen levels [7].

However, research on the application of whole-plant rape silage (including pod shells) in goats remains limited. Furthermore, although the nutritional value of rape straw is well established, most existing studies have focused on isolated performance metrics, failing to provide a systematic assessment along the integrated “intake–growth–slaughter–meat quality” continuum. The Chongming white goat is an indigenous breed from Shanghai and a member of the Yangtze River Delta white goat family. This small to medium-sized, multipurpose breed is valued for its fur, meat, and other products. Renowned for its strong adaptability and tender, flavorful meat with minimal gamey odor, it is highly popular among consumers in Shanghai [13]. Therefore, this study employed the Chongming white goat to evaluate the effects of replacing corn straw silage with whole-plant rape silage on key parameters including growth performances, carcass traits, meat quality, and gastrointestinal health (such as intestinal morphology and gut microbiota).

## 2. Materials and Methods

### 2.1. Silage Preparation

Forage rape (*Brassica napus* L.) was cultivated in experimental fallow fields at Shanghai Liquan Liangmu Agricultural Development Co., Ltd. (Shanghai, China) during late September 2023. Harvesting of whole-plant rape (including pod shells) was conducted at the one-week pod stage in late April 2024. The plants were subsequently air-dried for 72 h, achieving a dry matter content greater than 30%. Corn straw was collected after grain harvest and air-dried for 96 h. Both rape and corn straw were chopped into 2–5 cm segments using a forage cutter, thoroughly mixed with sucrose (addition ratio of 5 kg/t) and a bacterial inoculant blend (*Lactobacillus plantarum*, *Bifidobacterium* spp., *Bacillus subtilis*, *Enterococcus faecalis*, and effective microorganisms ≥1.0 × 10^9^ CFU/g; Guangxi Zhunong Livestock Technology Co., Ltd., Nanning, China), then compacted into polyethylene bag silos. The ensiling process was conducted for 60 days under controlled room temperature conditions (25 °C). The nutritional components of the corn straw silage and whole-plant rape silage are provided in the Supplementary Material Table S1.

### 2.2. Animals and Diets

Thirty-six 6-month-old male Chongming white goat lambs (average body weight 19.80 ± 2.59 kg) were randomly allotted to two dietary treatments (*n* = 18 per group). Each group was housed in three replicate pens (6 lambs per pen). The experimental goats were sourced from a breeding facility located in the Chongming District of Shanghai, China. They were housed in elevated pens measuring 4 m × 5 m (20 m^2^), with each pen accommodating six animals to ensure adequate space. The slatted flooring used for efficient manure drainage and contributed to maintaining hygiene within the pens. The barn environment was meticulously controlled, with a temperature range maintained between 22 and 26 °C, relative humidity set at 60–70%, and continuous mechanical ventilation to ensure optimal air quality. Each pen was equipped with a dedicated water trough and feeder, ensuring the lambs had ad libitum access to water and feed throughout the trial. The diets for both the control (CON) and treatment (TRT) groups consisted of 70% roughage and 30% concentrate. The roughage portion of the CON diet was 100% corn straw silage, while in the TRT diet, 50% of the corn straw silage was replaced with whole-plant rape silage (resulting in a 1:1 mixture). The lambs were offered feed twice per day (at 06:30 and 15:30). The trial included a 7-day adaptation phase prior to the 90-day data collection period. Complete details regarding the nutritional composition of the diets are presented in Supplementary Material Table S2.

### 2.3. Antioxidant Capacity Analysis

On the mornings of day 60 and day 90 of the experiment, fasting jugular venous blood samples (5 mL) were collected from all goats into heparin sodium-anticoagulated tubes. Plasma samples were separated by centrifugation at 3000× *g* for 15 min at 4 °C and stored at −20 °C until analysis. Assays for superoxide dismutase (SOD) and glutathione peroxidase (GSH-Px) activity, malondialdehyde (MDA) concentration, and total antioxidant capacity (TAC) were conducted using the respective commercial kits from Nanjing Jiancheng Bioengineering Institute (Nanjing, China), strictly following the supplier’s recommended procedures.

### 2.4. Growth and Slaughter Performances

During the experiment, goats were weighed monthly, and the feed offered was adjusted based on changes in body weight. Following the experiment, six goats per group (two per replicate) were randomly selected and subjected to a 24 h fast and a 2 h water deprivation. Immediately prior to slaughter, live weight was obtained. The goats were then humanely electrocuted and slaughtered at the slaughterhouse. After slaughter, the following components were removed from the goat: the hide, head (separated at the atlanto-occipital joint), portion distal to the carpal and tarsal joints, and internal organs (with the kidneys and perirenal fat retained). The remaining carcass was allowed to rest for 30 min and then weighed to obtain the carcass weight. The slaughter rate is calculated by dividing the sum of carcass weight and visceral fat weight (including omental and mesenteric fat) by the live weight at slaughter. Approximately 200 g *Longissimus dorsi* muscle was collected from each goat for amino acid composition and untargeted metabolomics analysis. Segments of the jejunum, ileum, and colon were also sampled. All samples were immediately frozen and stored at −20 °C for short-term preservation (up to one week) before being transferred to −80 °C freezers for long-term storage until analysis.

The pH determinations were carried out 45 min post-slaughter using a pH meter (Testo AG, Neustadt, Germany) equipped with automatic temperature compensation. Measurements were taken by inserting the probe approximately 2 cm into the *Semimembranosus* muscle at three random locations. Color measurement was conducted after a 45 min blooming period using a chroma meter (Konica Minolta, Tokyo, Japan) calibrated with a white standard plate. The meat color parameters (lightness (L*), redness (a*), and yellowness (b*) were measured on the *Longissimus dorsi* surface under the following settings: D65 light source, 10° observation angle, and 8 mm aperture. All measurements were performed in triplicate, and the average value was used for data analysis.

### 2.5. Amino Acid Composition Analysis

The amino acid profile of *Longissimus dorsi* muscle samples (*n* = 6) from Chongming white goats was determined by high-performance liquid chromatography (HPLC) (Waters Technology, Taunton, MA, USA). Briefly, approximately 100 mg of minced tissue was hydrolyzed in 6 M HCl (20 mL) at 110 °C for 22 h in sealed evacuated tubes. After automated pre-column derivatization, analysis was carried out on a Waters 2695 HPLC system (Milford, MA, USA) using a 10 µL injection volume. Chromatographic separation was achieved with mobile phase A (40 mM sodium dihydrogen phosphate, pH 7.8) and mobile phase B (acetonitrile/methanol/water, 45:45:10, *v*/*v*/*v*) at a flow rate of 1.0 mL/min and a column temperature of 37 °C. Identification and quantification were performed by comparing sample peak retention times and areas to those of known amino acid standards.

### 2.6. Untargeted Metabolomics

A total of 50 mg of muscle tissue was homogenized in liquid nitrogen, followed by the addition of pre-cooled methanol: water (2:5) to facilitate lipid extraction. After centrifugation at 3000× *g* for 15 min at 4 °C, the organic phase was collected and dried under a nitrogen stream. Lipid analysis was conducted using a UHPLC-Q Exactive HF-X system (Thermo, Vacaville, CA, USA) in both positive and negative ion modes. An Accucore C30 column (100 × 2.1 mm, 2.6 µm; Thermo, Vacaville, CA, USA) was employed for chromatographic separation at a constant temperature of 40 °C. A gradient elution program was run at a flow rate of 0.4 mL/min using two eluents: mobile phase A was 10 mM ammonium acetate in 50% acetonitrile (*v*/*v*) with 0.1% (*v*/*v*) formic acid, and mobile phase B was 2 mM ammonium acetate in a mixture of acetonitrile/isopropanol/water (10:88:2, *v*/*v*/*v*) with 0.02% (*v*/*v*) formic acid. Peak extraction, alignment, and normalization were performed using LipidSearch software (Version 5.1) (Thermo, Vacaville, CA, USA), and Principal component analysis (PCA) and Partial Least Squares Discrimination analysis (PLS-DA) through the R package ropls (Version 1.6.2). Related analyses were conducted on the Shanghai Meiji Cloud platform [14].

### 2.7. Morphology Determination

Intestinal segments from the jejunum, ileum, and colon were dissected and trimmed into 0.5 cm × 0.5 cm. Fixation was performed by immersion in 4% paraformaldehyde for 24 h. Post-fixation, samples were dehydrated, embedded in paraffin, and sectioned according to conventional histological protocols. After deparaffinization and H&E staining, morphological features were examined under a light microscope (Olympus VS200, OLYMPUS Corporation, Tokyo, Japan). Morphometric measurements of villus height, villus width, mucosal thickness, and crypt depth were determined.

### 2.8. 16S rRNA Sequencing and Data Processing

On day 60 of the experiment, fecal samples were collected from twelve randomly selected goats per group. Genomic DNA was isolated from fecal samples employing the QIAamp DNA Stool Mini Kit (Qiagen, Hilden, Germany) in accordance with the supplier’s protocol. DNA quality was assessed by measuring concentration on a NanoDrop ND-2000 spectrophotometer (Thermo Fisher, Vacaville, CA, USA) and confirming integrity via 1.5% agarose gel electrophoresis. Amplification of the bacterial 16S rRNA gene V3–V4 region was performed using the universal primers 338F and 806R. Following the construction of barcoded libraries, paired-end sequencing (2 × 300 bp) was conducted on an Illumina MiSeq platform (Illumina, San Diego, CA, USA), with all biological replicates (*n* = 12 per group) processed individually. The raw sequencing data have been deposited in the NCBI Sequence Read Archive under the accession number PRJNA1295007.

Following demultiplexing, raw sequencing reads were subjected to quality control processing using fastp (v0.19.6) for adapter trimming and quality filtering. Sequence assembly was subsequently performed with FLASH (v1.2.11) using default parameters. High-fidelity amplicon sequence variants (ASVs) were resolved through DADA2, which is implemented within the QIIME2 pipeline (2020.2 release), applying error-rate modeling to achieve single-nucleotide precision. To mitigate sequencing depth bias in diversity analyses, samples were rarefied to an even depth of 20,000 sequences per specimen, maintaining an average Good’s coverage of over 97.9%. Microbial functional profiles were inferred from ASVs using PICRUSt-based metagenome prediction.

Alpha diversity was estimated in Mothur version 1.30.1 by calculating the observed number of operational taxonomic units (OTUs), Chao1 richness, Shannon index, and Good’s coverage. The similarity of microbial communities among samples was visualized via principal coordinate analysis (PCoA) based on Bray–Curtis dissimilarity. This analysis, along with a PERMANOVA test to quantify the variance explained by the treatment and its statistical significance, was performed using the Vegan package (version 2.5-3) in R.

### 2.9. Statistical Analysis

Growth and slaughter performance, antioxidant capacity, amino acid profiles, and intestinal morphology were compared between the two groups using Student’s *t*-test in SPSS Statistics (version 19.0) (IBM Corp., Armonk, NY, USA). Statistical significance was defined as *p* < 0.05. A threshold of 0.05 ≤ *p* < 0.10 was defined as indicative of a trend. Data are expressed as mean ± standard error of the mean.

Significantly altered metabolites were identified by combining Variable Importance in Projection (VIP) scores from the Orthogonal Partial Least Squares-Discriminant Analysis (OPLS-DA) model with *p*-values from Student’s *t*-tests. Metabolites with a VIP > 1 and *p* < 0.05 were deemed statistically significant.

For microbial community analysis, differentially abundant bacterial taxa (from phylum to genus) were identified using Linear Discriminant Analysis Effect Size (LEfSe; http://huttenhower.sph.harvard.edu/LEfSe, accessed on 30 June 2024), with a significance threshold set at an LDA score > 2 and a *p*-value < 0.05.

## 3. Results and Discussion

### 3.1. Whole-Plant Rape Silage Does Not Adversely Affect the Growth and Slaughter Performance of Goats

As shown in Table 1, substituting 50% of corn straw silage with whole-plant rape silage over 30- and 60-day intervals did not significantly alter body weight between goat groups *(p* > 0.05). However, at 90-day interval the TRT group exhibited an increase in body weight (*p* = 0.067). Additionally, no significant differences in the average daily gain were found between the two groups at 30, 60, and 90 days *(p* > 0.05). After 90 days, carcass weight was significantly higher (*p* = 0.005) in TRT group compared to the CON group. Collectively, these results indicate that partial replacement of corn straw silage with whole-plant rape silage enhances growth at final periods of feedlot in goats.

Meat color is a critical quality indicator, with lower L*, higher a*, and lower b* values generally associated with superior appearance [15]. In this study, no significant differences *(p* > 0.05) were observed in L*, a*, b* or pH values between groups, a finding that is consistent with prior research [9].

### 3.2. Whole-Plant Rape Silage Relatively Improved Antioxidant Capacity of Goats

According to Table 2, no significant differences (*p* > 0.05) were observed in plasma SOD and GSH-Px activities between the groups after the 60-day feeding trial. In contrast, total antioxidant capacity (TAC) was significantly elevated in the TRT group (*p* < 0.01). Notably, by day 90, SOD and GSH-Px activities in TRT goats showed a potential, albeit non-significant, increasing trend compared to controls (*p* = 0.063 and *p* = 0.079, respectively).

Total antioxidant capacity (TAC) is a widely used parameter for assessing the overall antioxidant status of biological samples. It measures the cumulative response of antioxidants to free radicals generated under specific pathological conditions. The SOD and GSH-Px are essential antioxidant enzymes that work collaboratively to maintain redox homeostasis within cells [16]. These enzymes function synergistically to alleviate oxidative stress by neutralizing reactive oxygen species (ROS) and preventing cellular damage [17,18]. A reduction in the activity of both enzymes can lead to the accumulation of ROS, resulting in lipid peroxidation, protein denaturation, and DNA damage [17]. Consequently, SOD and GSH-Px activities serve as key biomarkers for evaluating oxidative stress levels.

The lack of significant differences in systemic antioxidant parameters among the dietary groups suggests that the partial replacement of corn silage with whole-plant rape silage was well-tolerated and did not elicit substantial oxidative stress in goats. Two non-exclusive hypotheses may explain this observation: firstly, the robust homeostatic capacity of the ruminant antioxidant system in response to moderate dietary changes [19]; and secondly, the potential that the nutritional disparities between the silages were not sufficiently pronounced to significantly impact systemic oxidative metabolism [20].

### 3.3. Whole-Plant Rape Silage Improved the Meat Quality of Chongming White Goats

In the present study, 21 types of amino acids were determined in the meat of each group, as shown in Table 3. Among these amino acids, there are nine essential amino acids, ten non-essential amino acids, and three semi-essential amino acids. However, no significant change (*p* > 0.05) was observed in the total amino acid content between the two groups. Dietary incorporation of whole-plant rape silage was associated with a significant reduction (*p* < 0.05) in the concentration of specific amino acids within muscle tissue relative to the CON group. The affected amino acids comprised five essential types, namely leucine, isoleucine, phenylalanine, methionine, and tryptophan, in addition to the semi-essential amino acid tyrosine and the non-essential amino acid asparagine anhydrous.

Amino acids, the fundamental structural units of proteins, critically determine meat’s flavor profile, textural properties, and nutritional quality. Research indicates that varying feeding conditions and dietary compositions can significantly impact the amino acid profile of meat, thereby affecting its overall quality [21]. Strategic modulation of silage botanical composition optimizes skeletal muscle fatty acid and amino acid profiles, thereby enhancing meat nutritional and sensory attributes. Furthermore, the use of alternative silage has demonstrated a positive effect on animal growth trait and muscle amino acid composition [22]. Substituting corn silage entirely with daylily silage did not significantly adversely affect the growth trait or muscle amino acid profiles of Tan sheep [22]. The utilization of this alternative silage feed can mitigate the scarcity of high-quality roughage resources and offer a viable feed alternative that does not compromise meat quality.

The taste properties of amino acids are strongly influenced by their hydrophobicity. A subset of amino acids characterized by low hydrophobicity—namely glycine, alanine, serine, and aspartic acid—are known for imparting a sweet taste, a property that leads to their classification as the sweet amino acids [22]. Conversely, amino acids characterized by high hydrophobicity predominantly produce a bitter flavor, referred to as bitter amino acids; examples include leucine, isoleucine, phenylalanine, tyrosine, tryptophan, histidine, and arginine [23]. Aspartic acid and glutamic acid are known as “umami amino acids” due to their key role in forming savory compounds. While the free acids taste sour, their sodium salts—sodium aspartate and monosodium glutamate (MSG)—are renowned for delivering the characteristic savory umami flavor [24,25]. This study found that feeding goats whole-plant rape silage significantly reduced the muscle content of bitter amino acids (e.g., leucine, isoleucine). This decrease suggests a potential pathway for flavor improvement. However, the small sample size (*n* = 6 per group) may have introduced substantial individual variation and wide confidence intervals for certain amino acids. Therefore, future studies should employ larger sample sizes to thoroughly investigate the impact of this dietary substitution on the overall flavor profile of goat meat and elucidate the underlying mechanisms.

**Figure 1:**
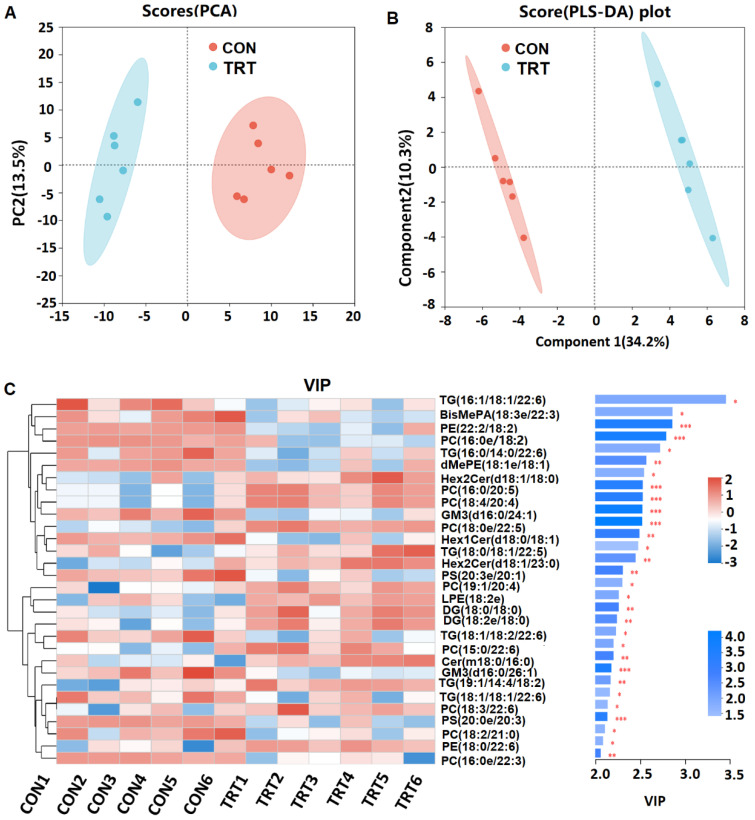
Effect of rape plant silage on lipid metabolism in goat muscle (*n* = 6). (**A**) Principal component analysis (PCA). (**B**) Partial Least Squares Discrimination analysis (PLS-DA). (**C**) Variable Importance in Projection (VIP) analysis. * *p* < 0.05, ** *p* < 0.01,*** *p* < 0.01. CON: goats fed corn straw silage; TRT: goats fed a diet containing a 1:1 mixture of whole-plant rape silage and corn straw silage. TG: triglyceride; PC: phosphatidylcholine; DG: diglyceride; TG: triglyceride; Hex1Cer: Simple Glc series; PE: phosphatidylethanolamine; PS: phosphatidylserine; dMePE: dimethylphosphatidylethanolamine; Hex2Cer: Simple Glc series; GM3: Gangliosides; Cer: Ceramides; LPE: lysophosphatidylethanolamine; BisMePA: Bis-methyl phosphatidic acid.

An analysis of the muscle lipidome was also conducted to assess the impact of dietary whole-plant rape silage. This investigation specifically targeted variations in the abundance of lipid species containing highly unsaturated fatty acids, which are pivotal compounds known to enhance the nutritional profile of muscle tissue. As illustrated in Figure 1A,B, significant differences in lipid molecule content were observed between groups. A total of 137 lipid molecules exhibited significant changes (VIP > 1, *p* < 0.05) following the introduction of whole-plant rape silage, among which 30 lipid molecules with VIP > 2 were subjected to further analysis. Among these 30 lipid molecules, there were 6 triglycerides (TG), 2 phosphoserines (PS), 2 phosphatidylethanolamines (PE), and 9 phosphatidylcholines (PC). The VIP value of TG (16:1/18:1/22:6) was 3.45, identifying it as the most significantly different lipid molecule in goat meat after feeding with whole-plant rape silage (Figure 1C). However, most differentially abundant lipid species did not show statistically significant fold-changes. The sole exception was phosphatidylcholine PC (18:0e/22:5), which exhibited a modest 1.2-fold increase (*p* < 0.05) in the muscle of TRT goats compared to controls (Supplementary Material Table S3). The absence of both significant fold-changes (>1.5) and statistical significance indicates that the dietary intervention with whole-plant rape silage did not substantially alter the muscle lipidome of goats.

### 3.4. Whole-Plant Rape Silage Promoted Intestinal Development

**Figure 2:**
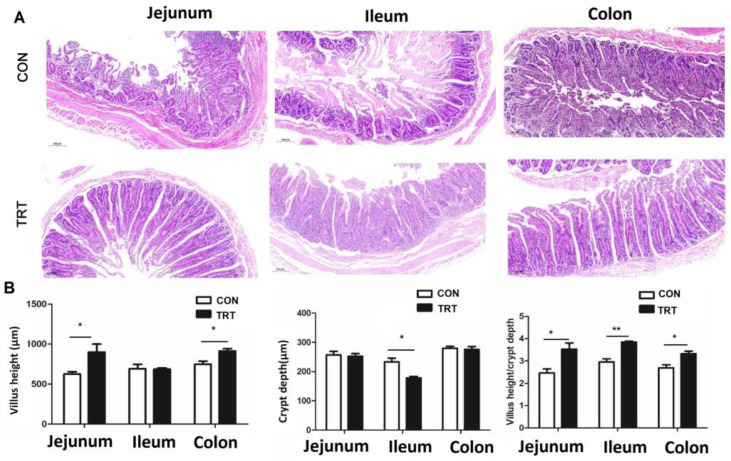
Effects of whole-plant rape silage on goat intestinal morphology (*n* = 6). (**A**) Hematoxylin and eosin (HE) staining analysis. Bar scale 200 μm. (**B**) Impact of the rape silage on the height of the villi, depth of the crypts, and the villus height to crypt depth ratio (VH/CD) in the gut of goat. * *p* < 0.05, ** *p* < 0.01. CON: goats fed corn straw silage; TRT: goats fed a diet containing a 1:1 mixture of whole-plant rape silage and corn straw silage.

The architecture of intestinal villi and crypts is essential for normal intestinal function. Research has established a direct correlation between intestinal morphology—specifically villus height and crypt depth—and the functional capacity of the gut, influencing both the renewal rate of epithelial cells and the efficiency of nutrient absorption [26]. An increase in villus height is typically associated with enhanced nutrient absorption efficiency, while the depth of crypts correlates with the proliferation and regenerative capacity of epithelial cells [27]. The depth of crypts determines the speed at which intestinal villi undergo mitosis to generate epithelial cells, reflecting the rate of cell generation. However, shallower crypts indicate an increase in cell maturation rate and enhanced secretion function [26]. Therefore, the villus height to crypt depth (VH/CD) ratio serves as an integrative measure, reflecting the small intestine’s digestive and absorptive capacity while providing a valuable benchmark for intestinal health status. Research has found that an increase in VH/CD often signifies improved intestinal health and enhanced absorption capacity [28]. Furthermore, research indicates that a higher VH/CD correlates significantly with improved intestinal morphology and functional capacity. This relationship is exemplified in a study that galactooligosaccharides intervention led to a marked increase in the duodenal VH/CD ratio in mice, resulting in significant improvements to intestinal integrity and immune modulation [29]. Our morphological analysis indicated that dietary intervention with whole-plant rape silage enhanced intestinal architecture in Chongming white goats (Figure 2A). The TRT group demonstrated superior villus structure, including increased colonic villus height (*p* < 0.05) and significantly reduced ileal crypt depth (*p* < 0.05), culminating in a higher (*p* < 0.05) VH/CD ratio compared to CON (Figure 2B). This suggests that whole-plant rape silage promotes villus development and optimizes intestinal morphology, which is likely to bolster intestinal barrier function and overall gut health.

### 3.5. Whole-Plant Rape Silage Regulated Gut Microbiota Composition

**Figure 3:**
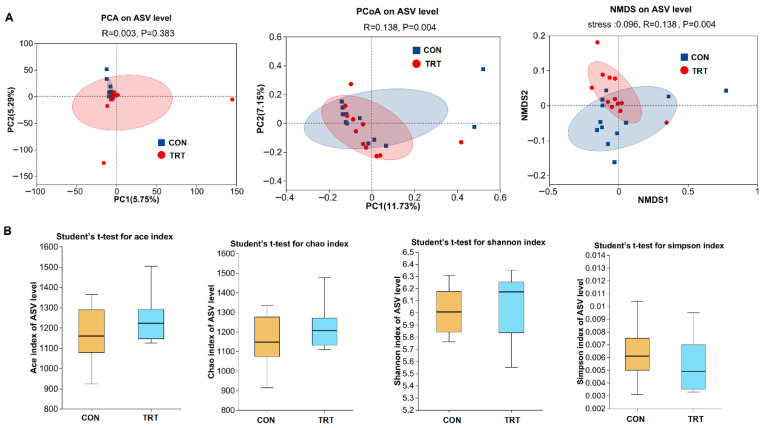
Comparison of fecal microbial community structures between whole-plant rape silage and corn straw silage treated goats (*n* = 12). (**A**) Beta diversity analysis. PCA, Principal component analysis; PcoA: principal Co-ordinates analysis; NMDS: non-metric multidimensional Scaling. (**B**) Alpha diversity analysis, and data represent mean values ±SEM. CON: goats fed corn straw silage; TRT: goats fed a diet containing a 1:1 mixture of whole-plant rape silage and corn straw silage.

Here, notable shifts in fecal microbiota composition were observed, particularly in beta diversity (Figure 3A, B). Principal Component Analysis, Principal Co-ordinates Analysis and Non-metric Multidimensional Scaling performed at the ASV level revealed clear separation between the CON and TRT groups (*p* < 0.01; Figure 3A). In contrast, alpha diversity analysis demonstrated that neither richness estimators (Chao and ACE) nor diversity indices (Shannon and Simpson) were significantly altered (*p* > 0.05; Figure 3B).

Improvements in intestinal structure are often linked to beneficial shifts in gut microbiota. Dietary interventions are increasingly recognized as a key approach to regulating gut microbiota, thereby influencing multiple aspects of human and animal health [30]. The modulation of gut microbiota via dietary approaches represents a viable strategy for enhancing health and preventing diseases. Studies have shown that incorporating garlic straw with corn silage improves dry matter digestibility and in vitro gas production, while also exerting beneficial effects on the rumen microbiota of Hu sheep [31].

**Figure 4:**
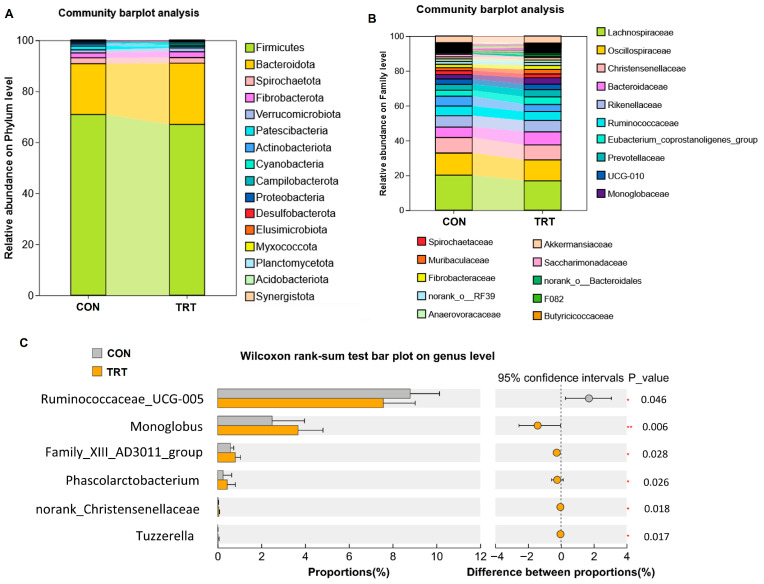
Taxonomic profiles of gut microbiota between control and treated goats (*n* = 12). (**A**) Relative abundance of the top 10 most abundant phyla in the gut microbiota. (**B**) Relative abundance of the top 10 most abundant genera in the gut microbiota. (**C**) Differential abundance of genera between CON and TRT goats. * *p* < 0.05, ** *p* < 0.01. CON: goats fed corn straw silage; TRT: goats fed a diet containing a 1:1 mixture of whole-plant rape silage and corn straw silage.

*Bacteroidota* and *Firmicutes* are two dominant phyla in the gut microbiota, with their proportional changes closely linked to host metabolism, immunity, and various diseases [32]. *Bacteroidota* plays a key role in breaking down complex polysaccharides like dietary fiber and generating short-chain fatty acids such as acetic acid and propionic acid [33]. This phylum contributes significantly to the regulation of host energy metabolism and the maintenance of immune homeostasis. A decrease in its abundance is associated with obesity and inflammatory bowel disease, particularly within the *Bacteroides* genus [33]. *Firmicutes* include various butyrate-producing bacteria (e.g., *Faecalibacterium prausnitzii*) and potential pathogens such as *Clostridium*. These bacteria contribute to energy extraction and may promote fat storage [32]. An elevated *Firmicutes*/*Bacteroidota* ratio is widely associated with metabolic disorders, including obesity and type 2 diabetes [34,35]. In the present study, dietary supplementation with whole-plant rape silage significantly increased the relative abundance of *Bacteroidota* and decreased that of *Firmicutes* at the phylum level (Figure 4A). At the genus level (Figure 4B), the abundance of *Lachnospiraceae* decreases, whereas that of *Bacteroidaceae* increases, suggesting that replacing 50% of corn straw silage with an equal amount of whole-plant rape silage may benefit the production of probiotics that degrade dietary fiber in the intestine.

In addition, LEfSe analysis revealed a significant reduction in the abundance of *Ruminococcaceae_UCG-005* (*p* = 0.046). Conversely, the relative abundances of *Phascolarctobacterium*, *Monoglobus*, and *Family_XIII_AD3011_group* were significantly increased (*p* < 0.05; Figure 4C).

*Ruminococcaceae_UCG-005*, a genus belonging to the family *Ruminococcaceae*, is functionally important within the gut microbiota of multiple animal species and contributes to host health and metabolic regulation. While it shows a positive correlation with beneficial traits like body weight and average daily gain [36], it is also associated with a higher incidence of diarrhea [37]. Dietary supplementation with insoluble fiber has been shown to increase the abundance of *Phascolarctobacterium*, a bacterial genus linked to various health benefits. This suggests a functional role for this microbe in fermenting dietary fiber and producing short-chain fatty acids, which contribute to gut health [38]. A study on the effects of quercetin, a polyphenolic compound, on atherosclerosis identified *Phascolarctobacterium* as a key microbiota signature, indicating its involvement in metabolic regulation and potential cardiovascular benefits [39]. Furthermore, *Phascolarctobacterium* has been implicated in the prevention of *Clostridioides difficile* infection by consuming succinate, thereby highlighting its contribution to gut homeostasis and protection against pathogen colonization [40].

*Family*_*XIII*_*AD3011_group*, although less studied, has been shown to be influenced by dietary components, particularly high-protein diets. Studies on high-meat-protein diets have shown an elevation in the abundance of *Family_XIII_AD3011_group*, which correlated with changes in tryptophan metabolism and the generation of metabolites like skatole and indole [41]. These results indicate that this bacterial group could play a role in the metabolic responses to high-protein dietary regimens, possibly influencing host physiology and overall health [41]. Additionally, *Monoglobus*, another noteworthy genus, has been less extensively studied [42]. Its functional significance in the gut ecosystem, however, can be extrapolated from research on taxonomically and functionally related microbes and their responses to dietary interventions [43]. The abundance and activity of *Monoglobus* are likely modulated by factors that also influence other fiber-degrading bacteria, suggesting a role in mediating the metabolic and health effects linked to dietary fiber consumption [44].

More specifically, we found that *Monoglobus*—which includes the genera such as *Acetobacter* and *Propionibacterium*—along with *Family_XIII_AD3011_group* (potentially involved in carbohydrate metabolism) and *Phascolarctobacterium* (which consumes lactate to produce propionate), all showed considerable enrichment (*p* < 0.05; Figure 4C). These shifts suggest a potential adaptation of the gut environment toward enhanced propionic acid metabolism and gut health.

## 4. Conclusions

The findings demonstrated that a 50% substitution of corn straw silage with whole-plant rape silage increased carcass weight and improved meat quality by reducing bitter amino acids (e.g., leucine, isoleucine). Furthermore, it enhanced intestinal morphology and promoted the growth of microbiota involved in fiber degradation (e.g., *Bacteroidaceae*), carbohydrate metabolism (e.g., *Family_XIII_AD3011_group*), and propionic acid metabolism (e.g., *Phascolarctobacterium*). These results indicate that whole-plant rape silage can be utilized as an alternative forage to improve carcass traits, meat quality, and gastrointestinal health in goats.

However, this study has limitations, including the absence of long-term data regarding the partial substitution of corn silage with whole-plant rape silage, the measurement of abdominal fat, omental and mesenteric fat, and a thorough analysis of goat meat flavor. To address these gaps, future studies should prioritize optimizing the substitution levels, assessing long-term effects, and elucidating the underlying mechanisms that enhance the flavor profile.

## Figures and Tables

**Table 1:** Effect of whole-plant rape silage on the growth and slaughter performance of goats.

Item	CON	TRT	*p*-Value
Initial BW (kg)	31.45 ± 0.87	33.19 ± 0.96	0.187
30 d BW (kg)	34.69 ± 0.99	36.13 ± 1.05	0.326
60 d BW (kg)	38.15 ± 0.88	39.47 ± 1.23	0.391
90 d BW (kg)	40.06 ± 1.07	43.04 ± 1.33	0.069
30 d ADG (g)	108.03 ± 11.45	97.84 ± 9.38	0.496
60 d ADG (g)	111.76 ± 8.77	104.61 ± 8.52	0.562
90 d ADG (g)	90.56 ± 8.56	109.41 ± 7.29	0.234
Carcass weight (kg)	21.01 ± 0.77 ^b^	25.72 ± 1.08 ^a^	0.005
Slaughter rate (%)	55.01 ± 0.52	54.4 ± 0.63	0.473
L*	3.29 ± 0.48	4.64 ± 0.69	0.141
a*	5.76 ± 0.28	6.2 ± 0.58	0.508
b*	4.45 ± 0.21	4.61 ± 0.48	0.773
pH	6.19 ± 0.08	5.89 ± 0.11	0.064

Note: ^a,b^ Data marked with different superscript letters are statistically significantly different (*p* < 0.05). Data are expressed as mean ± standard error of the mean. CON: goats fed corn straw silage; TRT: goats fed a diet containing a 1:1 mixture of whole-plant rape silage and corn straw silage; BW: body weight; ADG: average daily gain. *n* = 18 per group for growth performance analysis, and *n* = 6 per group for carcass and meat quality analysis.

**Table 2:** Effect of whole-plant rape silage on antioxidant capacity in plasma of goats (*n* = 18).

Time	Item	CON	TRT	*p*-Value
60 days	SOD (U/mL)	59.22 ± 1.94	62.44 ± 1.51	0.200
	GSH-Px (U/mL)	1206.68 ± 30.85	1260.58 ± 33.68	0.246
	TAC (nmol/mL)	0.95 ± 0.05	1.19 ± 0.04	0.000
	MDA (nmol/mL)	2.60 ± 0.19	2.38 ± 0.14	0.356
90 days	SOD (U/mL)	45.71 ± 1.73	50.23 ± 1.81	0.063
	GSH-Px (U/mL)	1010.84 ± 30.66	1099.57 ± 38.60	0.079
	TAC (nmol/mL)	0.32 ± 0.15	0.32 ± 0.02	0.887
	MDA (nmol/mL)	4.47 ± 0.39	4.97 ± 0.35	0.365

Note: CON: goats fed corn straw silage; TRT: goats fed a diet containing a 1:1 mixture of whole-plant rape silage and corn straw silage; SOD: superoxide dismutase; GSH-Px: glutathione peroxidase; MDA: malondialdehyde; TAC: total antioxidant capacity.

**Table 3:** Impact of whole-plant rape silage supplementation on goat muscle amino acid profiles (*n* = 6).

Category of Amino Acids		CON	TRT	*p*-Value
Non-essential amino acids	Alanine	1054.26 ± 7.03	1069.79 ± 15.55	0.384
	Asparagine Anhydrous	14.31 ± 2.49	8.05 ± 0.59	0.053
	Glutamine	7160.13 ± 2385.64	6239.03 ± 1646.68	0.757
	Glutamic Acid	8.74 ± 2.33	16.68 ± 4.57	0.153
	Serine	19.32 ± 2.23	14.71 ± 1.40	0.111
	Hydroxyproline	2.09 ± 0.23	2.37 ± 0.39	0.545
	Proline	15.90 ± 1.58	13.17 ± 0.67	0.141
	Cysteine	0.72 ± 0.29	0.29 ± 0.18	0.232
	Aspartate	8.56 ± 3.50	4.93 ± 3.34	0.471
	Subtotal	8284.04 ± 2392.64	7369.01 ± 1649.45	0.687
Semi-essential amino acid	Arginine	75.91 ± 13.01	77.26 ± 10.10	0.936
	Glycine	59.29 ± 6.97	61.58 ± 7.71	0.830
	Tyrosine	15.76 ± 0.42 ^a^	12.33 ± 0.48 ^b^	0.004
	Subtotal	150.96 ± 17.50	151.16 ± 13.89	0.565
Essential amino acids	Leucine	21.42 ± 1.10 ^a^	16.01 ± 1.21 ^b^	0.011
	Isoleucine	8.42 ± 0.24 ^a^	6.38 ± 0.48 ^b^	0.008
	Threonine	14.01 ± 1.40	9.62 ± 1.41	0.051
	Phenylalanine	8.36 ± 0.15 ^a^	6.42 ± 0.27 ^b^	0.000
	Lysine	141.85 ± 21.82	106.98 ± 15.87	0.225
	Histidine	126.71 ± 6.11	133 ± 12.81	0.667
	Methionine	4.85 ± 0.10 ^a^	3.58 ± 0.33 ^b^	0.007
	Valine	16.26 ± 0.88 ^a^	12.53 ± 0.89 ^b^	0.014
	Tryptophan	13.07 ± 0.65 ^a^	9.8 ± 1.03 ^b^	0.022
	Subtotal	354.94 ± 29.51	304.31 ± 18.57	0.111

Note: ^a,b^ Data marked with different superscript letters are statistically significantly different (*p* < 0.05). Data are expressed as mean ± standard error of the mean. CON: goats fed corn straw silage; TRT: goats fed a diet containing a 1:1 mixture of whole-plant rape silage and corn straw silage.

## Data Availability

The data presented in this study are openly available in NCBI Sequence Read Archive under the accession number PRJNA1295007.

## Supplementary Materials

The following supporting information can be downloaded at: https://www.mdpi.com/article/10.3390/foods14203512/s1, Supplementary Table S1. Nutrient composition of corn straw silage and rape silage. Supplementary Table S2. Feed composition and nutrient levels of goat diets. Supplementary Table S3. Effect of whole-plant rape silage supplementation on lipid metabolism in goat muscle (*n* = 6).
