# Supplementary material for: Whole-Plant Rape Silage-Based Diets for Chongming White Goats: An Integrated Assessment of Growth Performance, Meat Quality and Gut Microbiota
Source: Foods. 2025 Oct 15;14(20):3512. doi: 10.3390/foods14203512 (PMC12564722; doi:10.3390/foods14203512)
Supplement: Supplementary file 1 [file foods-14-03512-s001.zip › foods-3878302-supplementary.pdf]

Table S1. Nutrient composition of corn straw silage and rape silage.

Items	Corn Straw Silage	Rape Silage
Dry Matter (%)	24.2	30.2
Crude protein (%)	1.97	4.62
Crude fat (%)	0.36	0.51
Crude ash (%)	1.98	4.94
Acidity detergent fiber (ADF) (%)	8.22	11.31
Neutral detergent fiber (NDF) (%)	13.64	14.81
Lignin (%)	1.63	3.14
Total Sugar (%)	1.24	0.82
Calcium (%)	0.12	0.37
Phosphorus (%)	0.04	0.12
Magnesium (%)	0.07	0.08
Potassium (%)	0.33	1.36
Sodium (%)	0	0.11
Lactic acid	1.32	4.96
Acetic acid	0.33	0.32
pH	4.56	4.12

Table S2. Feed composition and nutrient levels of goat diets (air-dry basis).

Items	CON	TRT
**Ingredients**		
Corn	13.1	14.5
Soybean meal	10	9
Wheat bran	4.1	3.7
Corn Straw Silage	70	35
Rape Silage	0	35
NaCl	1.3	1.3
Premix [1]	1.5	1.5
Total	100	100
**Nutrient levels**		
Crude protein (%)	8.1	9.6
Acidity detergent fiber (ADF) (%)	25.33	29.35
Neutral detergent fiber (NDF) (%)	36.25	39.01
Calcium (%)	0.91	1.15
Phosphorus (%)	0.89	1.02

1 The premix provides the following per kg of diets: vitamin A 16,000 IU, vitamin D 5,000 IU, vitamin E 600 IU, Fe (as ferrous sulfate) 330 mg, Zn (as zinc sulfate) 260 mg, Cu (as copper sulfate) 60 mg, Mn (as manganese sulfate) 50 mg, I (as potassium iodide) 56 mg, Co 2.25 mg.

Table S3. Effect of whole-plant rape silage supplementation on lipid metabolism in goat muscle (n = 6).

Lipid	VIP _OPLS-DA	Fold Change (TRT/CON)	*p*-Value	Class name
PC (18:0e/22:5)	2.518	1.204	0.000	phosphatidylcholine
LPE (18:2e)	2.258	1.187	0.013	lysophosphatidylethanolamine
PE (18:0/22:6)	2.080	1.174	0.019	phosphatidylethanolamine
PE (18:1/22:5)	1.889	1.137	0.006	phosphatidylethanolamine
PE (16:1e/22:6)	1.950	1.130	0.005	phosphatidylethanolamine
CL (24:0/18:2/20:3/24:1)	1.652	1.124	0.002	Cardiolipin
PS (18:0/22:4)	1.974	1.122	0.000	phosphatidylserine
PS (20:2/20:3)	1.616	1.115	0.042	phosphatidylserine
PC (18:1/18:3)	1.657	1.100	0.002	phosphatidylcholine
PE (17:0/18:2)	1.640	1.099	0.009	phosphatidylethanolamine
TG (18:0/18:1/22:5)	2.472	1.095	0.037	triglyceride
PC (18:0/18:0)	1.686	1.094	0.001	phosphatidylcholine
PS (18:0/22:5)	1.674	1.094	0.001	phosphatidylserine
PG (16:1/18:2)	1.501	1.093	0.023	phosphatidylglycerol
PS (20:3e/20:5)	1.570	1.093	0.011	phosphatidylserine
PE (16:0/20:5)	1.368	1.087	0.032	phosphatidylethanolamine
dMePE (16:0/20:3)	1.566	1.086	0.003	dimethylphosphatidylethanolamine
MGDG (20:2e/22:5)	1.593	1.084	0.005	Monogalactosyldiacylglycerol
Hex2Cer (d18:1/18:0)	2.538	1.082	0.016	Simple Glc series
PE (18:1/22:2)	1.429	1.081	0.017	phosphatidylethanolamine
dMePE (16:1/18:1)	1.529	1.079	0.004	dimethylphosphatidylethanolamine

CL (21:1/20:4/16:0/18:1)	1.356	1.071	0.031	Cardiolipin
dMePE (16:1e/16:1)	1.305	1.071	0.033	dimethylphosphatidylethanolamine
Hex2Cer (d18:1/23:0)	2.445	1.066	0.005	Simple Glc series
Cer (d18:1/19:0)	1.469	1.064	0.003	Ceramides
PE (20:4/20:4)	1.229	1.062	0.023	phosphatidylethanolamine
PE (18:1/20:5)	1.223	1.062	0.032	phosphatidylethanolamine
PC (19:1/20:4)	2.298	1.062	0.018	phosphatidylcholine
PC (18:3/22:6)	2.131	1.061	0.024	phosphatidylcholine
PE (18:0/22:4)	1.494	1.060	0.001	phosphatidylethanolamine
dMePE (16:0/16:0)	1.165	1.058	0.048	dimethylphosphatidylethanolamine
PE (18:0/20:5)	1.426	1.058	0.001	phosphatidylethanolamine
PC (18:4/20:4)	2.524	1.057	0.001	phosphatidylcholine
PC (16:0/20:5)	2.524	1.057	0.001	phosphatidylcholine
dMePE (17:0/18:2)	1.187	1.057	0.044	dimethylphosphatidylethanolamine
PS (20:4/21:0)	1.349	1.057	0.002	phosphatidylserine
MePC (16:0e/20:4)	1.943	1.056	0.033	Methyl phosphatidylcholine
CL (23:0/16:0/18:0/20:4)	1.221	1.056	0.019	Cardiolipin
PC (15:0/22:6)	2.198	1.054	0.012	phosphatidylcholine
DG (18:2e/18:0)	2.235	1.052	0.004	diglyceride
PS (20:0/20:4)	1.110	1.052	0.036	phosphatidylserine
DG (18:0/18:0)	2.256	1.051	0.002	diglyceride
TG (19:1/14:4/18:2)	2.165	1.051	0.008	triglyceride
Hex2Cer (d13:0/18:2)	1.256	1.051	0.003	Simple Glc series
Cer (m18:0/16:0)	2.197	1.051	0.002	Ceramides
dMePE (15:0/18:2)	1.120	1.046	0.015	dimethylphosphatidylethanolamine
PI (18:0/22:4)	1.068	1.046	0.049	phosphatidylinositol

CL (21:0/16:0/18:0/20:4)	1.387	1.046	0.000	Cardiolipin
PE (17:0/20:4)	1.036	1.046	0.028	phosphatidylethanolamine
PE (18:1e/20:5)	1.392	1.045	0.000	phosphatidylethanolamine
PS (19:0/20:4)	1.324	1.044	0.001	phosphatidylserine
PC (16:1/20:4)	1.324	1.044	0.001	phosphatidylcholine
PG (20:0/20:4)	1.217	1.043	0.000	phosphatidylglycerol
PC (16:1e/16:1)	1.077	1.040	0.029	phosphatidylcholine
dMePE (16:0/20:5)	1.217	1.040	0.000	dimethylphosphatidylethanolamine
CL (21:0/18:0/18:0/22:4)	1.231	1.039	0.001	Cardiolipin
PC (20:3/22:6)	1.788	1.038	0.016	phosphatidylcholine
PS (18:2/20:4)	1.011	1.038	0.015	phosphatidylserine
PE (16:0/20:4)	1.090	1.035	0.007	phosphatidylethanolamine
DG (18:0/16:0)	1.953	1.034	0.000	diglyceride
PC (15:0/20:4)	1.773	1.033	0.014	phosphatidylcholine
PC (20:0/16:0)	1.795	1.033	0.011	phosphatidylcholine
PC (18:1e/17:1)	1.016	1.031	0.013	phosphatidylcholine
PC (11:0/22:4)	1.616	1.029	0.020	phosphatidylcholine
PC (16:0/22:6)	1.720	1.029	0.010	phosphatidylcholine
PC (18:1/13:0)	1.429	1.027	0.048	phosphatidylcholine
DG (18:2e/20:5)	1.507	1.026	0.008	diglyceride
PC (18:3e/19:0)	1.391	1.023	0.047	phosphatidylcholine
PC (16:0/20:4)	1.015	1.023	0.000	phosphatidylcholine
Cer (d16:0/20:0+O)	1.530	1.022	0.001	Ceramides
Cer (d16:0/18:0+O)	1.353	1.020	0.013	Ceramides
Cer (t18:0/16:0)	1.406	1.020	0.011	Ceramides
PC (18:0/20:5)	1.303	1.020	0.037	phosphatidylcholine

Cer (t18:0/18:0)	1.311	1.019	0.019	Ceramides
PC (14:1e/22:6)	1.256	1.018	0.023	phosphatidylcholine
PE (18:0p/20:5)	1.282	1.018	0.034	phosphatidylethanolamine
BisMePA (18:3e/20:4)	1.280	1.018	0.035	Bis-methyl phosphatidic acid
Cer (d16:0/18:0)	1.354	1.018	0.021	Ceramides
Cer (d16:0/16:0)	1.432	1.017	0.005	Ceramides
PE (18:0/18:1)	1.171	1.017	0.048	phosphatidylethanolamine
DG (18:0/18:1)	1.175	1.016	0.042	diglyceride
PC (14:0e/18:1)	1.093	0.988	0.021	phosphatidylcholine
PC (8:0e/21:0)	1.072	0.987	0.014	phosphatidylcholine
PC (8:1e/23:0)	1.094	0.987	0.032	phosphatidylcholine
Cer (t18:1/18:0)	1.179	0.984	0.032	Ceramides
PC (14:0e/16:0)	1.379	0.983	0.002	phosphatidylcholine
PC (12:0e/18:1)	1.226	0.982	0.047	phosphatidylcholine
TG (18:1/18:1/22:4)	1.476	0.982	0.001	triglyceride
Cer (t17:0/18:0)	1.289	0.980	0.034	Ceramides
Cer (d18:2/24:1)	1.348	0.977	0.045	Ceramides
PC (8:0e/23:0)	1.458	0.974	0.023	phosphatidylcholine
PE (16:1e/18:1)	1.150	0.973	0.000	phosphatidylethanolamine
TG (18:0/18:1/22:6)	1.652	0.968	0.021	triglyceride
PE (16:1e/22:4)	1.126	0.967	0.007	phosphatidylethanolamine
PC (18:1e/20:3)	1.072	0.967	0.009	phosphatidylcholine
TG (16:0/18:1/22:6)	1.678	0.966	0.048	triglyceride
Cer (t18:0/19:0)	1.604	0.966	0.041	Ceramides
PE (18:0/18:2)	1.147	0.965	0.006	phosphatidylethanolamine
Pet (22:0/22:6)	1.027	0.964	0.026	phosphatidylethanol

TG (18:1/18:1/22:1)	1.971	0.959	0.020	triglyceride
PG (20:0/18:2)	1.246	0.949	0.006	phosphatidylglycerol
PS (20:3e/20:4)	1.427	0.948	0.003	phosphatidylserine
TG (18:1/18:1/22:6)	2.158	0.947	0.026	triglyceride
PS (20:4e/22:4)	1.137	0.947	0.039	phosphatidylserine
TG (18:1/18:2/22:6)	2.225	0.946	0.013	triglyceride
Cer (d18:1/21:0)	1.288	0.940	0.035	Ceramides
SM (d18:1/24:0)	1.697	0.937	0.000	sphingomyelin
PS (20:3e/22:3)	1.498	0.936	0.001	phosphatidylserine
Hex1Cer (t17:0/22:3)	1.531	0.934	0.004	Simple Glc series
PC (16:1e/17:1)	1.503	0.934	0.007	phosphatidylcholine
PIP2 (18:2e/23:0)	1.366	0.934	0.010	phosphatidylinositol
PC (16:1e/14:0)	1.448	0.934	0.005	phosphatidylcholine
PS (20:4e/18:1)	1.385	0.933	0.030	phosphatidylserine
PE (19:0/20:4)	1.303	0.932	0.029	phosphatidylethanolamine
PS (20:3e/22:5)	1.437	0.925	0.019	phosphatidylserine
PC (18:0/17:0)	1.486	0.925	0.014	phosphatidylcholine
PS (18:0/20:4)	1.717	0.922	0.000	phosphatidylserine
MGDG (20:2e/22:4)	1.791	0.918	0.000	Monogalactosyldiacylglycerol
PS (18:0/18:1)	1.589	0.917	0.014	phosphatidylserine
TG (16:0/14:0/22:6)	2.717	0.915	0.016	triglyceride
PE (18:1e/16:0)	1.543	0.913	0.036	phosphatidylethanolamine
PG (16:1/18:1)	1.617	0.912	0.010	phosphatidylglycerol
BisMePA (18:3e/22:3)	2.857	0.903	0.015	Bis-methyl phosphatidic acid
PS (20:4e/20:4)	1.652	0.901	0.025	phosphatidylserine
PS (20:3e/16:0)	1.623	0.895	0.022	phosphatidylserine

PE (18:1e/22:2)	1.911	0.884	0.005	phosphatidylethanolamine
PC (16:0e/22:3)	2.054	0.879	0.002	phosphatidylcholine
PS (20:0e/20:3)	2.131	0.876	0.001	phosphatidylserine
GM3 (d16:0/26:1)	2.175	0.872	0.000	Gangliosides
TG (16:1/18:1/22:6)	3.453	0.867	0.013	triglyceride
PS (20:3e/20:1)	2.302	0.842	0.002	phosphatidylserine
PC (18:2/21:0)	2.101	0.840	0.020	phosphatidylcholine
dMePE (18:1e/18:1)	2.564	0.826	0.004	dimethylphosphatidylethanolamine
GM3 (d16:0/24:1)	2.520	0.823	0.000	Gangliosides
PC (16:0e/18:2)	2.786	0.823	0.000	phosphatidylcholine
Hex1Cer (d18:0/18:1)	2.487	0.820	0.001	Simple Glc series
PE (22:2/18:2)	2.854	0.801	0.000	phosphatidylethanolamine
